# Pexophagy: The Selective Degradation of Peroxisomes

**DOI:** 10.1155/2012/512721

**Published:** 2012-03-27

**Authors:** Andreas Till, Ronak Lakhani, Sarah F. Burnett, Suresh Subramani

**Affiliations:** Section of Molecular Biology, Division of Biological Sciences, University of California, San Diego, La Jolla, CA 92093-0322, USA

## Abstract

Peroxisomes are single-membrane-bounded organelles present in the majority of eukaryotic cells. Despite the existence of great diversity among different species, cell types, and under different environmental conditions, peroxisomes contain enzymes involved in **β**-oxidation of fatty acids and the generation, as well as detoxification, of hydrogen peroxide. The exigency of all eukaryotic cells to quickly adapt to different environmental factors requires the ability to precisely and efficiently control peroxisome number and functionality. Peroxisome homeostasis is achieved by the counterbalance between organelle biogenesis and degradation. The selective degradation of superfluous or damaged peroxisomes is facilitated by several tightly regulated pathways. The most prominent peroxisome degradation system uses components of the general autophagy core machinery and is therefore referred to as “pexophagy.” In this paper we focus on recent developments in pexophagy and provide an overview of current knowledge and future challenges in the field. We compare different modes of pexophagy and mention shared and distinct features of pexophagy in yeast model systems, mammalian cells, and other organisms.

## 1. Introduction to Peroxisome Biology

Peroxisomes were initially described as “microbodies” in a Ph.D. thesis on the cellular morphology of rodent kidneys [1] and were characterized as novel eukaryotic organelles by De Duve and Baudhuin in the 1960s [2]. Biochemical analysis of isolated peroxisomes from rat liver resulted in the identification of several enzymes involved in hydrogen peroxide generation and detoxification and thus led to the term “peroxisome” for this new organelle. Almost 50 years later, despite significant insights regarding peroxisome function, several aspects of peroxisome biology still remain unresolved. This is partly based on the fact that peroxisomes display an unusually high variability in function, morphology, and biochemical features. For example, the presence of enzymes involved in the glyoxylate cycle has resulted in the denotation “glyoxysomes” for some plant peroxisomes [3], while the same organelle is dubbed “glycosome” in trypanosomatids because it houses glycolytic enzymes [4, 5]. Exemplifying the remarkable specialization of peroxisomal enzymes is the protein luciferase and proteins required for synthesis of penicillin. Luciferase is responsible for the bioluminescent characteristic of the firefly *Photinus pyralis* [6, 7] and the enzymatic cascade involved in penicillin production derives from the fungus *Penicillium chrysogenum* and its relatives [8, 9]. In vertebrates, peroxisomes harbor the enzymatic pathways for synthesis of specialized ether phospholipids vital for integrity of the central nervous system [10].

**Figure 1:**
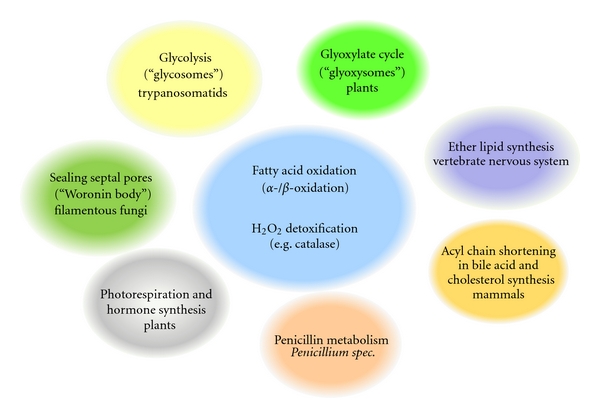
Overview of peroxisome functions in different organisms and tissues. Peroxisomes display a great variety in metabolic pathways as defined by their respective enzymatic content. Most eukaryotes share peroxisomal enzymes for fatty-acyl-CoA metabolism (*α*- and *β*-oxidation) and detoxification of hydrogen peroxide by catalase. In addition, several specialized metabolic pathways housed in the peroxisomal matrix of various organisms or tissues are shown.

In contrast to these specializations, most peroxisomes share the enzymatic components for the *β*-oxidation of fatty-acyl-CoA derivatives, as well as for the production and degradation of hydrogen peroxide and other reactive oxygen species (ROS). The common evolutionary origin of all peroxisome subtypes is best illustrated by the ubiquitous presence of orthologs of a specific set of *PEX* genes, encoding peroxins, involved in peroxisome biogenesis, maintenance, and division. Additional commonalities are that all peroxisomal proteins are encoded in the nucleus, translated in the cytosol and imported into the peroxisomes by a highly conserved set of localization signals (called peroxisomal targeting signals or PTSs) and corresponding receptors and transporters [11, 12].  Figure 1 summarizes shared and unique metabolic and enzymatic functions of peroxisomes.

The evolutionary origin of peroxisomes is still a matter of debate [13]. Their presence in all main eukaryotic taxa and the mentioned similarities argue for a singular evolutionary origin in a common ancestor of eukaryotic cells, most likely as a consequence of an increase in oxygen levels in the archaic atmosphere. While it was initially hypothesized that peroxisomes evolved in the course of events related to endosymbiosis, similar to mitochondria and plastids [14–16], research in the past decade has provided conclusive evidence that peroxisomes are not remnants of endosymbiotic microorganisms but have evolved from specialization of distinct parts of the endoplasmatic reticulum (ER) [17, 18]. Peroxisomes (unlike mitochondria and chloroplasts) have a single membrane, do not possess their own genome, and require several peroxisomal membrane proteins (PMPs) that transit via the ER before reaching their final destination in the peroxisomal membrane [19–22]. In the light of these findings it is generally believed that peroxisomes represent organelles originating from a specialization of the endomembrane system, rather than examples of endosymbiotic events. 

The vital importance of peroxisomes in higher eukaryotes is documented by the dramatic effects of peroxisome dysfunction on human health. Peroxisomal disorders (PDs) are subdivided into two major groups: “single peroxisomal enzyme/transporter deficiencies” (PEDs) and “Peroxisomal Biogenesis Disorders” (PBDs). PEDs are caused by a functional defect in one peroxisomal pathway and include metabolic syndromes such as acatalasia, Acyl-CoA deficiency and X-linked adrenoleukodystrophy [23]. PBDs are caused by mutations affecting a set of at least 12 human genes, which function in peroxisome biogenesis and assembly (*PEX* genes), resulting in manifestation of numerous pathological conditions [24, 25]. PBDs involve autosomal recessive neurodevelopmental disorders that display numerous other symptoms including skeletal and craniofacial dysmorphism, liver dysfunction, and retinopathy. These diseases are caused by complete or partial loss of peroxisome functionality and include the Zellweger syndrome spectrum disorders (e.g., Zellweger syndrome, neonatal adrenoleukodystrophy, and infantile Refsum's disease) as well as rhizomelic chondrodysplasia punctata [26, 27]. 

The severity of these defects emphasizes the pivotal role of peroxisomal metabolism for cellular integrity, especially in neuronal cells. In line with these observations, peroxisomes serve an important function in the central nervous system for the formation and maintenance of the myelin sheath and for the preservation of long-term axonal integrity [10, 28, 29]. In addition, recent reports point to a specific role of peroxisomal metabolism as a determinant of the cellular aging process, with peroxisome-derived ROS being triggers of antiaging pathways (at low concentrations), but also being decisive accelerators of aging by damage accumulation (at high concentrations) [30, 31]. This is further underscored by the finding that aging human fibroblasts accumulate peroxisomes with impaired protein import capacity, leading to ROS accumulation and exacerbation of the aging process [32]. Homeostasis in peroxisome number and functionality not only plays a role in the described disease settings, but also for the physiological aging process.

## 2. Peroxisome Homeostasis

**Figure 2:**
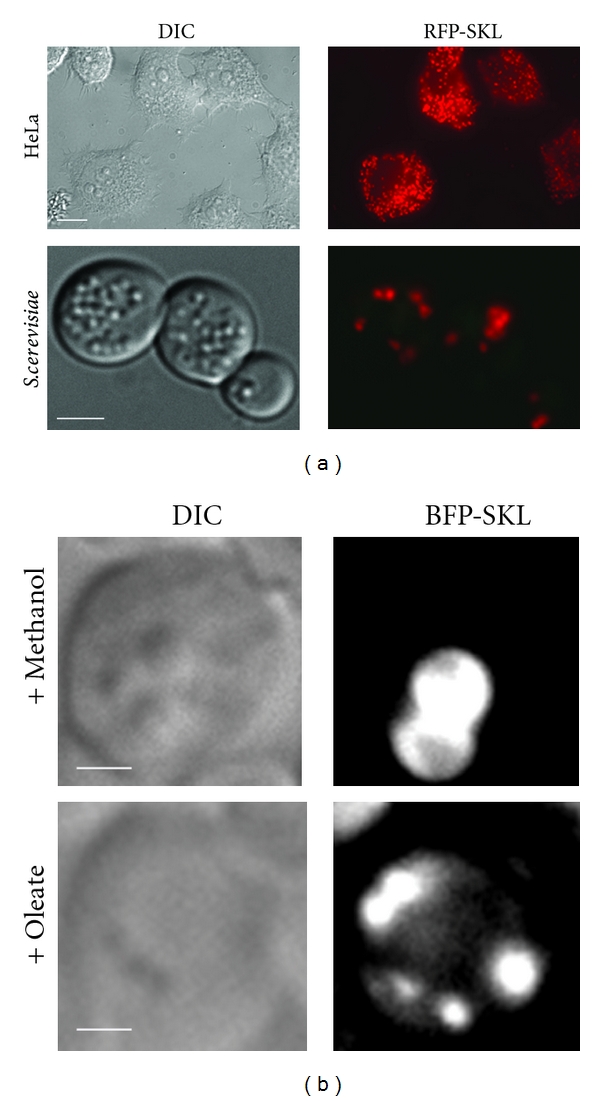
Comparison of peroxisome number and morphology in different eukaryotic cells and under different proliferation conditions. (a) Upper panel: Human HeLa cells expressing the peroxisomal marker, RFP-SKL, under basal growth conditions. Lower panel: *S. cerevisiae* cells expressing RFP-SKL after peroxisome induction in oleate medium. The relative number of peroxisomes per cell differs greatly between different eukaryotic cell types. Size marker = 2 *μ*m. (b) Grayscale images of *P. pastoris* cells expressing BFP-SKL as peroxisomal marker. Upper panel: large, clustered methanol-induced peroxisomes; lower panel: small, unclustered oleate-induced peroxisomes. Note the difference in size and appearance of peroxisomes induced by different carbon sources. Size marker = 2 *μ*m.

Due to their importance for a variety of metabolic functions, peroxisome number is tightly controlled by environmental conditions. Yeasts (e.g., *Hansenula polymorpha, Pichia pastoris, and Saccharomyces cerevisiae*), are capable of utilizing different carbon sources and increasing peroxisome number and biomass when grown in these media requiring peroxisomal metabolism (Figure 2). Conversely, the shift of these cells from peroxisome induction conditions to carbon sources wherein peroxisomes are unnecessary triggers the degradation of superfluous peroxisomes by autophagy. These observations of peroxisome induction and removal have resulted in utilization of yeasts as model organisms to study peroxisome biogenesis, and turnover, which have led to the identification of several genes and mechanisms controlling peroxisome homeostasis [11, 33–47]. In rodents, the administration of phthalate esters or hypolipidemic drugs such as fibrates results in upregulation of peroxisomal proteins and a concomitant increase in peroxisome number [48]. This process is dependent on members of a special class of nuclear receptors, called “peroxisome proliferator-activated receptors (PPARs)” [49, 50]. However, this effect does not represent a general conserved mechanism since PPAR agonists fail to induce peroxisome proliferation in human cells [51, 52]. In contrast, it has been demonstrated that drugs, such as 4-phenylbutyrate (4-PBA), that act as chemical chelators and/or affect histone deacetylase (HDAC) activity can act as nonclassical peroxisome proliferators independent of PPAR activity in human cells [53, 54].

Here we present model systems to study peroxisome turnover and outline mechanisms that contribute to peroxisome homeostasis by regulating the selective degradation of peroxisomes. The main focus will be on selective degradation of peroxisomes in the vacuolar/lysosomal compartment, a process mediated by components of the general autophagy core machinery and usually referred to as pexophagy. 

## 3. Methylotrophic Yeasts as Model Systems for Pexophagy

The large peroxisome clusters of methylotrophic yeasts (e.g., *P. pastoris* and *H. polymorpha*), as well as the experimental ease of manipulation of peroxisome number, volume, and content by media shifts in a genetically tractable organism, have facilitated studies on pexophagy. These yeasts, when grown in media containing methanol as the sole carbon source, rapidly proliferate their peroxisomes, which can occupy up to 40% of the cell volume. This makes fluorescence imaging of tagged proteins involved in pexophagy, as well as biochemical analysis of peroxisomal markers, much easier to monitor than in mammalian or other yeast systems. The autophagic degradation of peroxisomes was first noted by De Duve and Baudhuin [2] when they observed the appearance of peroxisomes within the lysosomes of mammalian cells, thus documenting the earliest description of pexophagy. Since then, much has been learnt from studies on pexophagy conducted in methylotrophic yeasts. 

## 4. Modes of Pexophagy: Micropexophagy and Macropexophagy

All organisms from yeast to humans possess basal and inducible macroautophagy. During macroautophagy (referred to here as “autophagy”), a double membrane originates from a site known as the phagophore assembly site (PAS) to engulf cargo into a double-membrane vesicle known as the autophagosome, which upon fusion with a lysosome (or vacuole in yeast cells), releases into the lysosomal/vacuolar lumen an autophagic body comprised of a single membrane surrounding the cytosolic cargo. Once in the lysosomal lumen, the membrane and other macromolecular contents of the autophagic body are degraded by hydrolases to their constituent building blocks for reuse by the cell. This entire process, from the assembly of the PAS, to the engulfment of cargo into autophagosomes, fusion of autophagosomes with the lysosome/vacuole and subsequent degradation of the cargo, is orchestrated by the hierarchical recruitment of autophagy-related (Atg) proteins [80]. An alternative process called microautophagy also exists, in which the lysosome/vacuole membrane invaginates to engulf cytosolic cargo directly to degrade and recycle it [81, 82].

**Figure 3:**
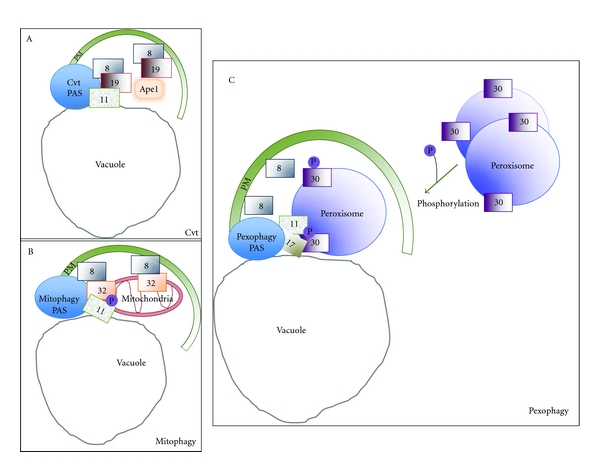
Similarities and differences between selective autophagy pathways. Various selective autophagy pathways share similar molecular mechanisms. They require a receptor that interacts with the cargo, recruits a scaffold protein (Atg11) that organizes the core autophagic machinery at the PAS, and mediates recruitment of Atg8, which initiates phagophore elongation from the PAS. In the Cvt pathway (a) Atg19 and Atg34 are the receptors for the cargo proteins aminopeptidase I (Ape1) and alpha-mannosidase, respectively. These receptors bind to Atg11 at the Cvt-specific PAS to initiate membrane expansion of the phagophore. (b) The mitophagy-specific phagophore membrane expansion from the PAS is initiated by Atg32, a mitochondrial outer membrane protein. Atg32 also interacts with Atg11 and Atg8. (c) The pexophagy receptor, Atg30, is localized at the peroxisome membrane, via interaction with the PMPs, Pex3, and Pex14. It is phosphorylated upon induction of pexophagy resulting in interaction of Atg30 with core autophagic machinery components, Atg11 and Atg17. In the case of pexophagy, the direct Atg8 interaction partner is still unknown.

In contrast to the nonselective nature of cargo engulfed by macroautophagy and microautophagy, other autophagy-related pathways capture cargo selectively from the cytosol. These include oligomeric proteins delivered to the vacuole by the cytosol-to-vacuole targeting (Cvt) pathway, ribosomes (ribophagy), and subcellular organelles such as peroxisomes (pexophagy), mitochondria (mitophagy), parts of the ER (ER-phagy), and segments of the nucleus (micronucleophagy) [38]. In most of these selective processes, the phagophore membrane, originating from specific PAS structures required for each form of selective autophagy (e.g., Cvt- or pexophagy-specific PAS, Figures 3(a) and 3(c)), engulfs the specific cargo and delivers it to the lysosome/vacuole for degradation. The source of the phagophore membrane is a widely debated topic within the field of autophagy, with the focus primarily on how Atg9 (ATG9L1 in mammals), which is the only transmembrane protein of the core Atg machinery, is recruited to the PAS [83]. Atg9 is thought to be involved directly or indirectly in trafficking membrane and/or lipid components during phagophore expansion from the PAS. However, this mechanistic concept remains a hypothesis [84–86].

To date, 35 autophagy-related (*ATG*) genes involved in several autophagy-related pathways have been discovered. Macropexophagy and micropexophagy (described next) are both used in *P. pastoris* for selective peroxisome degradation [87, 88]. As these represent specialized types of autophagy, it should not be surprising that they require many of the core genes also used for autophagy as well as specific genes in addition (see Table 1) [38, 89]. Many yeast mutants with pexophagy defects provide insights into the mechanism of the two pexophagy modes (see Table 1).

**Figure 4:**
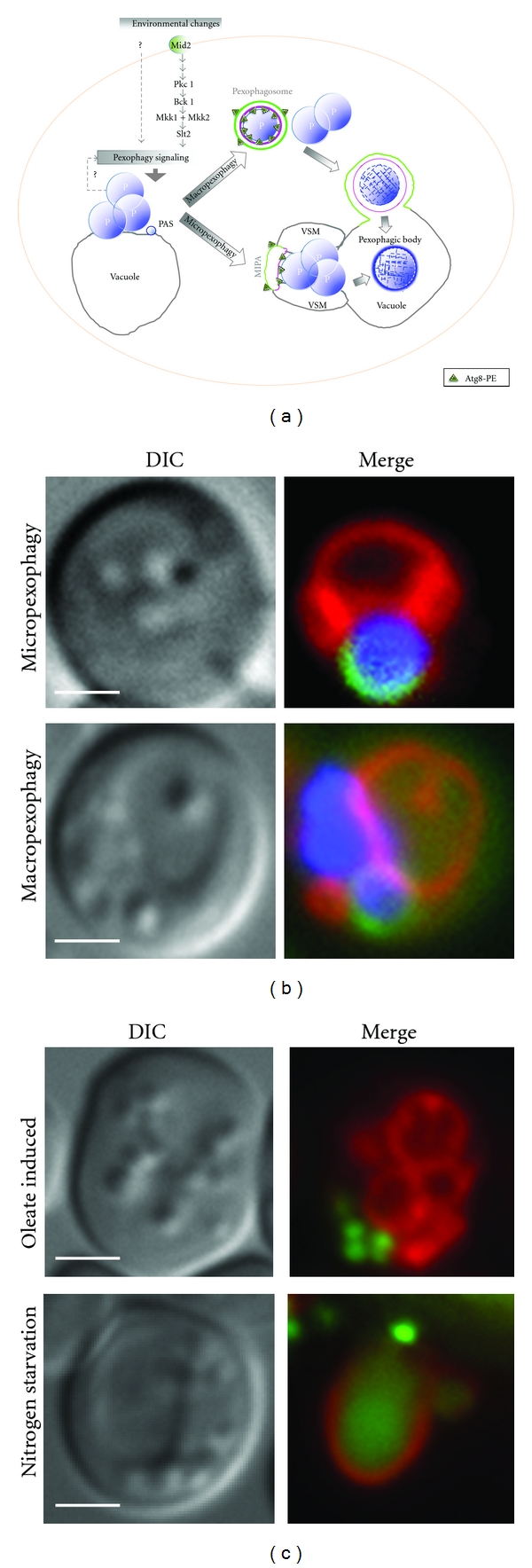
Micropexophagy and macropexophagy. (a) Micropexophagy differs from macropexophagy in vacuole dynamics and formation of the MIPA instead of the pexophagosome. A pexophagy-specific PAS, required for both forms of pexophagy, is characterized by its localization near the peroxisome and also touching the vacuolar membrane. Micropexophagy can target a peroxisome cluster for degradation by vacuole remodeling to form cup-like vacuolar sequestration membranes (VSMs) and a lid-like cover called the MIPA (micropexophagy-specific membrane apparatus). Macropexophagy is characterized by individual sequestration of targeted peroxisomes into a pexophagosome, followed by its fusion with the vacuole for degradation and recycling. Pexophagy signaling is dependent on Mitogen-activated protein kinase (MAPK) pathways (Mid2-Slt2 cascade), but may also be triggered by internal (unknown) factors, including signals related to the status of, or metabolic need for, (e.g., damaged or superfluous) peroxisomes. (b) The upper panel depicts a single *P. pastoris* cell that has undergone peroxisome induction (in methanol) and has then been switched to micropexophagy conditions (glucose). The vacuole (red, FM 4–64) is shown surrounding the targeted peroxisome cluster (blue, BFP-SKL). The MIPA (green, GFP-Atg8) forms a lid over the cup-like VSMs. The lower panel illustrates pexophagosome formation around a single peroxisome under macropexophagy conditions (ethanol). (c) *S. cerevisiae* cell labeled with GFP-tagged thiolase (a peroxisome matrix marker) and vacuole marker (FM 4–64, red) shows proliferated peroxisomes under nutrient-rich conditions (in oleate, top panel). When the cells are switched to glucose without nitrogen, peroxisomes are targeted to the vacuole by macropexophagy and GFP accumulates in the vacuole (lower panel).

Micropexophagy occurs when a cluster of peroxisomes is directly engulfed by vacuolar sequestering membranes (VSMs) that extend from a septated vacuole, and a double-membrane structure called the micropexophagy-specific membrane apparatus (MIPA) [60]. The MIPA extends from the PAS to form a cup-shaped lid over the VSM-engulfed peroxisomes and fuses with the VSMs to completely sequester the targeted peroxisomes from the cytosol and to ultimately deliver the pexophagic body into the vacuole lumen to be degraded by resident vacuolar enzymes (Figure 4(a)). During macropexophagy, an individual peroxisome is surrounded by the phagophore membrane originating from the pexophagy-specific PAS to form a double membrane-bounded pexophagosome (Figure 4(a)), before the outer membrane fuses with the vacuole membrane in a process resembling macroautophagy [41]. In *P. pastoris*, the choice between induction of either micro- or macropexophagy is determined by ATP levels in the cell [90]. High levels of ATP induce micropexophagy while lower levels activate macropexophagy. One explanation for this observation may be that the massive vacuolar rearrangement during micropexophagy and formation of the MIPA may be a more energy-intensive process than formation of the pexophagosome and thus may demand more energy in the form of ATP from the cell.

## 5. Nutrient Conditions That Induce Pexophagy

In *S. cerevisiae*, pexophagy is induced by transferring cells from growth media containing oleate as a carbon source to glucose-containing medium without a nitrogen source [91]. In *P. pastoris*, peroxisomes can be induced when cells are grown in media containing methanol, oleate, or amines. Transferring cells grown in methanol to ethanol or from oleate or methylamine to glucose without nitrogen induces macropexophagy (Figures 4(b) and 4(c)) [92]. Shifting cells from methanol medium to glucose induces micropexophagy (Figures 4(b) and 4(c)) [93]. Intriguingly, the two modes of pexophagy can be triggered by different experimental conditions in different yeasts. In *H. polymorpha*, macropexophagy, rather than micropexophagy, is induced when cells are shifted from methanol medium to glucose [47]. 

Interestingly, it was shown that simultaneous treatment of *H. polymorpha* with both nitrogen limitation and excess glucose conditions results in concomitant induction of both microautophagy and macropexophagy, thus exemplifying the fact that selective (i.e. pexophagy) and nonselective (autophagy) pathways can be initiated in parallel [94].

## 6. Regulation of Yeast Pexophagy

It has long been realized that not only surplus, but also damaged components or potentially toxic structures within the cytosol of eukaryotic cells can be selectively removed by autophagy. Using ectopic expression of a temperature-sensitive degron-Pex3 fusion, it was recently shown in *H. polymorpha* that damage to peroxisomes by abruptly removing the essential PMP, Pex3, causes pexophagy to occur [71]. This conditional selective degradation was apparent even when cells were placed in conditions that would normally require peroxisome biogenesis for cell growth. In methanol-excess conditions the authors saw a transient increase of ROS in wild-type cells that corresponds with the degradation of the peroxisome matrix protein, alcohol oxidase, as well as PMPs, Pex3, and Pex14, suggesting the possible physiological significance of pexophagy. However, it is unclear at present if there is a similar requirement of Pex3 removal from the peroxisome membrane for pexophagy in other methylotrophic yeasts such as *P. pastoris*, where Pex3 is actually essential to recruit the pexophagy receptor, Atg30, to the peroxisome, before the organelle is targeted for pexophagy. 

The signaling events that regulate the specific removal of cellular components are still poorly understood. The emerging role of intracellular signaling pathways controlling pexophagy was shown by our group and has since been replicated and refined further. Using the degradation of a peroxisomal marker to investigate the role protein kinases play in pexophagy in *S. cerevisiae*, the Slt2 mitogen-activated protein kinase (MAPK) and several other upstream components of this signaling pathway were shown to be required for pexophagy, but not for pexophagosome formation, suggesting a block at the step of pexophagosome targeting or pexophagosome-vacuole fusion [37].

**Figure 5:**
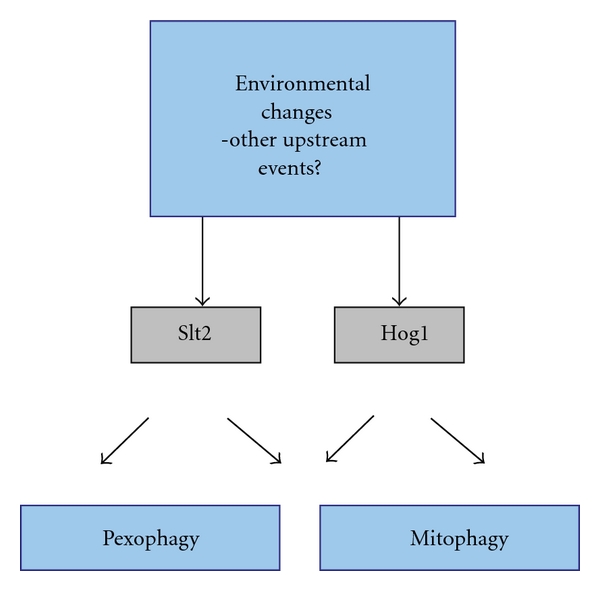
Signal transduction cascades regulating selective autophagy in yeast. Mitogen-activated protein kinase (MAPK) cascades contribute to differential regulation of selective autophagy pathways. As recently shown, the Slt2 and Hog1 signal transduction pathways regulate both mitophagy and pexophagy [37, 96]. Besides the obvious role of environmental factors such as nutritional conditions, details of other upstream events are poorly understood.

This theme of the involvement of yeast MAPK in selective autophagy has been extended by the recent discovery that Slt2 also plays a role in mitophagy [95], along with another MAPK (Hog1) (Figure 5) [95, 96]. Slt2 is crucial for recruiting mitochondria to the PAS, a step required for the specific packaging of cargo into autophagosomes. Interestingly, mitophagy in mammalian cells is activated by ERK2, another MAPK [97]. Thus, the differential involvement of MAPK pathways represents a central process in controlling diverse selective autophagy pathways [95].

Other signaling pathways have also been shown to have a direct role in pexophagy [35, 77, 98]. The phosphoinositide, phosphatidylinositol-3-phosphate (PtdIns3P), as well as the sole phosphatidylinositol 3-kinase, Vps34, that generates PtdIns3P in yeast, are required for all autophagy-related pathways, including pexophagy [36, 99]. In addition, phosphatidylinositol-4-phosphate (PtdIns4P), as well as the kinase that is responsible for PtdIns4P generation (Pik1) and Atg26, a sterol-glucosyltransferase that binds PtdIns4P via its GRAM domain, are necessary for micropexophagy in *P. pastoris* [46].

## 7. General Themes of Selective Autophagy Pathways

Since all autophagy-related pathways share common components required for PAS assembly, elongation of the phagophore membrane around cargo, vesicle formation, fusion and vacuolar degradation, the key decision point in any selective autophagy pathway is the mechanism by which the core autophagy machinery is redirected to degrade primarily selective cargo. The study of these selectivity factors for pexophagy in yeast has revealed a set of key principles. Where applicable, we describe how these events are relevant to other selective autophagy pathways.

1. Every selective autophagy pathway studied to date requires a specific cargo receptor. Examples of these include Atg30 for pexophagy [34], Atg19 and Atg34 for the Cvt pathway [100–102], and Atg32 for mitophagy [103, 104].
2. These cargo receptors typically have a tripartite role in (a) cargo binding, (b) interaction with Atg11, a protein required by all selective yeast autophagy pathways to create the specific PAS structures from which the phagophore membrane will expand [100, 103, 104], and (c) interaction with Atg8, via an Atg8-interaction motif (AIM) [105], to allow phagophore expansion [100, 103, 104]. The receptors Atg19 and Atg32, required for the Cvt and mitophagy pathways in yeast, have all these properties, but as of now, only the first two roles have been attributed to Atg30 during pexophagy [34].
3. The selective autophagy receptors are often synthesized even under conditions wherein the cargoes are not degraded, but receptor activation often relies on protein modifications, such as phosphorylation or ubiquitination [34, 96, 106].
4. Some of the pexophagy-mediating factors, such as Atg11 and the sterol glucosyltransferase Atg26 that binds PtdIns4P [46], are required in an absolute fashion for the degradation of large cargoes, but are partially dispensable when the cargo size is small [107]. We predict that since the phagophore membrane has to engulf cargoes of varying sizes from individual cytosolic proteins to organelles, bacteria and viruses, analogous factors will be required for selective autophagy of other large cargoes.
5. Specialized membrane structures, such as the MIPA, are needed for micropexophagy, and not for macropexophagy. Indeed, the protein Atg35 is needed for MIPA formation during micropexophagy, but not for pexophagosome formation during macropexophagy [43].
6. Generally the receptors are degraded in the vacuole along with the cargo.

## 8. The Pexophagy-Specific PAS

Like autophagy, pexophagy is also initiated at a specific PAS (Figure 3(c)) that is distinct from other types of PAS for selective autophagy (Figures 3(a) and 3(b)). The autophagy-specific PAS is organized by Atg11, Atg17, Atg29, and Atg31, but Atg11 is dispensable [108]. The Cvt-specific PAS requires Atg11 and Atg19 for its organization [100–102] (Figure 3(a)), whereas the mitophagy-specific PAS uses Atg11 and Atg32 [103, 104] (Figure 3(b)). The pexophagy-specific PAS is organized by Atg11, Atg17, and Atg30 [34, 107].

For the onset of pexophagy in *P. pastoris*, Atg30 phosphorylation by a hitherto unknown kinase occurs and facilitates direct physical interaction with Atg11 [34]. The two proteins colocalize at the PAS, and Atg30 also directly interacts with Atg17. The roles of Atg11 and Atg17 are as scaffolds at the PAS that recruit other proteins, such as constituents of the core autophagy machinery described next. Surprisingly, there is a size requirement of the scaffolding proteins. For degradation of small peroxisomes, Atg11 and Atg17 are only partially required, but are essential for degradation of large peroxisomes in nitrogen-starvation conditions [107].

The assembly of a specific PAS is followed by the recruitment of core proteins of the autophagic machinery to the PAS including, but not limited to Atg1, Atg2, Atg5, Atg8, Atg9, Atg12, Atg13, Atg16, Atg18, Atg23, Atg24, Atg25, Atg27, Atg28, Atg35, and the PtdIns3-kinase (PI3K) complex. These proteins typically assemble in a complex hierarchy [109], such as our demonstration that the recruitment of PtdIns-3-Kinase to the PAS precedes Atg8 recruitment [78].

## 9. Elongation of the Phagophore Membrane

The protein Atg35 is a micropexophagy-specific protein recruited by Atg28 and is required for efficient MIPA formation but not for pexophagosome formation, giving the first evidence that the formation of the MIPA could be genetically distinct from the formation of the pexophagosome in macropexophagy [43].

Oku et al. [44] discovered that Atg26, a sterol glucosyltransferase that synthesizes sterol glucoside, is essential for pexophagy, but not autophagy in *P. pastoris*. They showed that the protein is associated with the MIPA during micropexophagy, and that a single amino acid substitution within the GRAM domain (domain found in glycosyltransferases, Rab-like GTPase activators, and myotubularins) of the protein abolished this association [44]. However, it was found that although Atg26 is required for utilization of decane in *Y. lipolytica*, it was unnecessary for pexophagy in this yeast, showing that sterol glucosyltransferase play different functional roles in the two yeasts [68]. 

In *P. pastoris*, phosphatidylinositol-4-phosphate (PtdIns4P) initiates *de novo* membrane synthesis that is required for pexophagy. PtdIns4P, generated primarily by the PtdIns-4-kinase, Pik1, recruits Atg26 via its GRAM domain [46], and the sterol glucosyltransferase activity of Atg26 at the nucleation complex is necessary for the elongation of the membrane.

In both *S. cerevisiae* and *P. pastoris*, the only integral membrane protein of the autophagy machinery, Atg9, cycles between a peripheral compartment comprising a reservoir of Atg9 and the PAS, or PAS-like structures. The shuttling mechanism has been studied in both organisms but the process is better understood in *S. cerevisiae* and is therefore described next, before the role of this protein in pexophagy is described.

In *S. cerevisiae*, Atg9 colocalizes at the PAS but is not present on completed autophagosomes, suggesting it must be recycled during autophagosome formation. It cycles between a peripheral compartment and the PAS [85]. The anterograde trafficking of Atg9 from the peripheral compartment to the PAS requires Atg11, Atg23, and Atg27 [86]. Atg9 retrieval from the PAS is regulated by the Atg1-Atg13 signaling complex and requires Atg2, Atg18, and the PtdIns3P generated by the Atg14-containing PtdIns-3-kinase complex [110]. However, only Atg2, Atg18, and PtdIns3P are necessary for Atg9 recycling, while the Atg1-Atg13 complex and Atg1 kinase activity, but not Atg2, Atg18, and PtdIns3P, are necessary for Atg23 cycling to and from the PAS [110].

The subcellular movement of Atg9 in *S. cerevisiae* requires interaction with the actin cytoskeleton as has been shown by the sensitivity of relocation of Atg9 to the inhibitor Latrunculin A, as well as by the phenotype displayed by conditional mutants of actin and the actin-related protein Arp2 [111, 112].

The proteins Atg11 and/or Atg17 are necessary for Atg9 recruitment to the PAS [84, 113]. Also required at the PAS is PtdIns3P, generated by the Vps34 (PtdIns-3-kinase) complex, to recruit PtdIns3P-binding proteins (e.g., Atg18 and Atg24), which then recruit yet other proteins, such as Atg2, to the PAS [65].

*P. pastoris* Atg9 (*Pp*Atg9) is necessary for the formation of the VSM, assembly of the MIPA, and for pexophagosome formation. As in *S. cerevisiae*, the *P. pastoris* Atg9 also shuttles to the PAS from a peripheral compartment, perhaps supplying the membrane to the PAS and elongating the phagophore membrane to form the VSM, MIPA, and pexophagosome [63]. *Pp*Atg9 shuttles from a peripheral compartment near the ER/mitochondria to unique perivacuolar structures (PVS; PAS-like structures) that contain Atg11, but not Atg2 or Atg8. Atg9 then traffics from the vacuole surface to the VSMs that engulf peroxisomes for degradation [63]. Movement of the PpAtg9 from the peripheral compartment to the PVS requires PpAtg11 and PpVps15 (a subunit of the PtdIns-3-kinase). PpAtg2 and PpAtg7 are essential for PpAtg9 trafficking from the PVS to the vacuole and sequestering membranes, whereas trafficking of PpAtg9 proceeds independent of PpAtg1, PpAtg18, and PpVac8. How exactly PpAtg9 contributes to the formation of the MIPA and pexophagosome formation is less clear.

In *P. pastoris*, expression of dominant-negative forms (Sar1-T34N and Sar1-H79G) of the ER protein Sar1, impairs Atg8 lipidation and MIPA formation, but not the formation of the VSMs or the trafficking of Atg11 and Atg9 to these VSMs during micropexophagy [70]. During macropexophagy, the expression of Sar1-T34N inhibited the formation of the pexophagosome, whereas Sar1-H79G suppressed the delivery of the peroxisome from the pexophagosome to the vacuole. In this case, the pexophagosome contained Atg8 in wild-type cells, but in cells expressing Sar1-H79G these organelles contain both Atg8 and endoplasmic reticulum components, suggesting a defect in retrieval of components back to the ER, prior to pexophagosome/vacuole fusion.

The protein Atg25 has been described in *H. polymorpha* to be required for macropexophagy. It interacts with Atg11 and colocalizes with it at the PAS. In its absence, peroxisomes are constitutively degraded by nonselective microautophagy, a process that in wild-type *H. polymorpha* is only observed under nitrogen starvation conditions, suggesting that nonselective microautophagy is deregulated in *H. polymorpha atg*25Δ cells [39]. 

## 10. Requirement of Specific Proteins during the Final Stages of Pexophagy

Atg24, a molecule with a PtdIns3P-binding module (PX domain), is required for micropexophagy and macropexophagy, but not for general autophagy in *P. pastoris* and *S. cerevisiae* [33]. CFP-tagged PpAtg24 localizes to the vertex and boundary region of the pexophagosome-vacuole fusion complex during macropexophagy. Depletion of PpAtg24 blocked macropexophagy after pexophagosome formation and before its fusion to the vacuole. These results suggest that PpAtg24 is involved in the regulation of membrane fusion at the vacuolar surface during pexophagy via binding to PtdIns3P and could potentially be involved in pexophagosome fusion with the vacuole [33]. During micropexophagy, *Ppatg*24Δ cells form the MIPA and exhibit aberrantly septated vacuoles, reminiscent of other mutants defective in vacuolar fusion, but engulfment of peroxisomes is also impaired [33]. 

## 11. Pexophagy in Mammalian Cells

**Figure 6:**
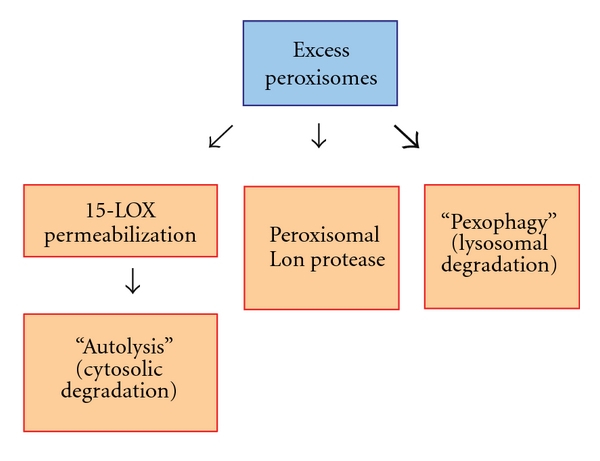
Peroxisome degradation pathways in mammalian cells. Surplus peroxisomes or their contents (e.g., peroxisomal matrix proteins) can be degraded by at least three distinct mechanisms: Lon protease-mediated proteolysis, 15-lipoxygenase (15-LOX)-mediated cytosolic degradation (autolysis), and pexophagy (autophagy-mediated lysosomal degradation). Current studies suggest that the majority of peroxisomes are degraded by pexophagy (indicated by bold arrow).

In contrast to yeast models, which have greatly contributed to the mechanistic understanding of pexophagy as outlined above, the molecular details of mammalian pexophagy are less well understood. This is partly based on fundamental differences between mammalian and yeast peroxisomes. While in yeasts the number of peroxisomes varies between 1–20 dependent on the species and growth conditions (see above), average mammalian cells contain between several hundred to thousands of peroxisomes (Figure 2(a)) [114]. Induction of peroxisome proliferation in rodents by phthalate esters [e.g., di-(2-ethylhexyl)phthalate; DEHP)], hypolipidemic drugs (e.g., fibrates) or nonclassical peroxisome proliferators (e.g., 4-PBA) results in a 2-3-fold increase of peroxisomal mass, which is a significantly smaller effect compared to the effects observed in yeasts. Consequently, quantitative analyses of peroxisome turnover in mammalian systems are limited by the detection method applied. Mammalian peroxisomes differ from those of yeast cells not only in number and induction mechanisms, but also by their modes of selective degradation. At least three independent degradation systems have been described: the Lon protease system, 15-lipoxygenase (15-LOX)-mediated autolysis and lysosomal degradation/pexophagy (Figure 6) [115]. Based on studies using *Atg7* conditional knockout mice it is estimated that up to 20–30% of the mass of liver peroxisomes is degraded by Lon protease-mediated mechanisms and 15-LOX-mediated autolysis of peroxisomes, whereas the remaining 70–80% are destroyed by autophagic mechanisms [115].

The peroxisomal isoform of the Lon protease is an ATP-dependent protease with chaperone-like activity that is involved in degradation of misfolded and unassembled peroxisomal proteins. Lon protease is upregulated in rats under peroxisome proliferation conditions (e.g., administration of DEHP) and further increases its levels after withdrawal of the inducing drug while peroxisomal enzymes are quickly degraded [116]. Subsequently, Lon protease activity catalyzes the breakdown of proteins resident in the peroxisomal matrix, indicating that it contributes to the reduction of peroxisome mass, if not quantity. Interestingly, the yeast ortholog of the Lon protease (encoded by the *PLN *gene in *H. polymorpha*) appears to be essentially involved in peroxisome quality control mechanisms, with only about a 25% contribution to reduction of peroxisome numbers, which increased only slightly from 2.6/cell to 3.3/cell in the absence of Pln, whereas the peroxisome number increased to 5.4/cell in the absence of the *ATG1* gene required for all forms of autophagy [117]. Assuming that the Lon protease has similar roles in yeast and mammals, it is conceivable that relative to autophagic mechanisms, it plays a relatively modest role (in the range of 25%) in reducing peroxisome number.

The cytosolic enzyme, 15-LOX, can associate with peroxisomal membranes leading to localized membrane disruption [118]. Structural breakdown subsequently exposes the peroxisomal content to cytosolic proteases resulting in its rapid degradation. This pathway appears to be initiated in parallel to pexophagy after drug-mediated accumulation of peroxisomes and accounts for removal of a limited fraction of excess peroxisomes.

While the abovementioned pathways contribute partially to peroxisome homeostasis under certain cellular conditions and other data argue for a role of the proteasome system by undefined mechanisms [119], the vast majority of selective peroxisome degradation is mediated by autophagosomal-lysosomal processes resembling yeast macropexophagy. As mentioned, early reports from the 1970s already noted the selective lysosomal degradation of mitochondria and peroxisomes during the diurnal cycle in rat inner organs [120], but it was only shown later that the autophagy machinery is specifically involved in degradation of surplus peroxisomes in mouse liver [121]. This was demonstrated by comparing abundance and degradation efficiency of peroxisomes after treatment with phthalate ester for 2 weeks and chase after drug removal one week later in wild-type and autophagy-deficient Atg7^−/−^ mice. The salient findings of this study emphasize mechanistic similarities to the above-mentioned yeast models used to study pexophagy: environmental conditions that require peroxisomal enzymes (e.g., oleate/methanol for yeasts, chemical peroxisome proliferators for rodents) lead to peroxisome proliferation, followed by pexophagic degradation when the organelles are no longer required or can be used as a resource for alternative pathways. This biological theme of a metabolic switch involving adaptation to changing external factors and thereby triggering pexophagy is also reminiscent of organelle remodeling in pathogenic fungi and parasitic protozoa as will be outlined in the next section. 

A detailed functional analysis of peroxisome degradation using an *in vitro* cell culture system showed for the first time that peroxisomes are preferentially degraded over cytosolic proteins under starvation/recultivation conditions [119]. This study used Chinese Hamster Ovary (CHO) cells to describe autophagy-mediated peroxisome turnover when switching culture conditions from starvation in Hank's solution to reconstitution in nutrient-rich medium. The authors show convincingly that the peroxisomal membrane protein, Pex14, is bound by autophagosome-anchored LC3-II (i.e., the processed and lipidated form of LC3) under starvation conditions. Pex14 is an essential component of the peroxisomal translocon complex, which facilitates import of cytosolic proteins into the peroxisomal matrix. It is noteworthy that the dual role of Pex14 for both peroxisome assembly and selective degradation has also been shown for yeast systems [47]. Moreover, the study by Hara-Kuge and Fujiki points to an involvement of the cytoskeleton in this process by demonstrating the requirement of intact microtubules for the LC3-II/Pex14 interaction [119]. As an intriguing example of the competitive nature of the processes involved, binding of Pex14 to either LC3-II or the peroxisomal import receptor, Pex5, proved to be mutually exclusive. This might point to a general mechanism that ensures functional segregation of metabolically active and degradation-prone organelles. Although this study uses an unusual experimental setup by applying starvation followed by recultivation in rich medium, it opens the avenue for future studies addressing the question of how exactly PMPs contribute to physical interactions with the autophagy machinery. In line with these observations, a recent study describes the role of a Rab7-effector protein, FYCO1 (FYVE and coiled-coil domain-containing 1), as the physical link between LC3 family members, PtdIns3P and microtubule plus end-directed transport [122, 123], but the exact role of this mechanism for pexophagy in particular has not been addressed yet.

The dynamics of peroxisome turnover in mammalian cells under normal cultivation conditions have nicely been addressed in a recent publication [124]. Using HaloTag-labelled peroxisomal marker proteins to follow the long-term fate of peroxisomes in cultured CHO cells and mouse fibroblasts, the authors show that mammalian peroxisomes have a half-life of approximately 2 days under normal cultivation conditions and that peroxisomes of different age display a different capacity to import newly synthesized proteins. In addition, this study shows that even under normal growth conditions, pexophagy contributes to the majority of turnover of this organelle as demonstrated by sensitivity to 3-methyl adenine (3-MA, an autophagy inhibitor) treatment. These findings emphasize the dual role of autophagy-related pathways: while autophagy principally serves to ensure nutrient recycling under starvation conditions, the same machinery fulfills the purpose of a quality control and homeostasis mechanism even in the presence of all nutrients.

**Figure 7:**
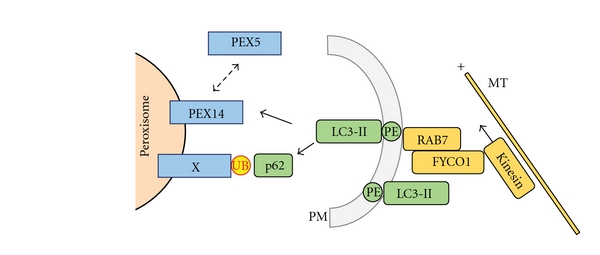
Hypothetical mechanistic model of pexophagy in mammalian cells. Processed and lipidated LC3 (LC3-II) is integrated into the expanding phagophore membrane (PM) and also may be involved in facilitating directed movement of the PM structure by interacting with microtubules (MT) via the RAB7 effector FYCO1 and motor protein Kinesin. Targeting of peroxisomes may either be accomplished by p62-mediated detection of ubiquitin (UB) motifs on still unknown peroxisomal membrane (or membrane associated) proteins (X) or by direct binding of LC3 to PEX14, a process which is discussed to compete with the binding of PEX5 to PEX14 (dotted arrow). See text for details.

Because the autophagy machinery in mammalian cells targets ubiquitinated protein aggregates, experiments were designed to address whether monoubiquitination of peroxisomal proteins could cause the autophagic clearance of peroxisomes [125]. Using overexpression of PMPs, Pmp34 and Pex3, fused on the cytosolic side to a ubiquitin variant genetically tailored to block polyubiquitination, it was found that exposure of a single ubiquitin moiety on the cytosolic face of the peroxisomal membrane was sufficient to trigger turnover of this organelle. Specificity of this affect was demonstrated by analyzing sensitivity to protein topology and to the autophagy inhibitor, 3-MA, thus confirming the requirement of the autophagy machinery in degradation of the ubiquitin-labeled peroxisomes. Moreover, the study showed that the ubiquitin-binding autophagy adaptor, p62, is involved in selective degradation of peroxisomes under the chosen conditions. Although this study is primarily based on overexpression of ectopic proteins and the artificial placement of a ubiquitin tag on the peroxisomal membrane and does not identify the physiological target of this process, it has some interesting implications. The general requirement of p62 for mammalian peroxisome homeostasis was demonstrated even in the absence of ectopic ubiquitin tagging, since knockdown of p62 significantly increased endogenous peroxisome numbers under the experimental conditions. Furthermore, of all mammalian autophagy adaptors identified so far (e.g., p62/SQSTM1, NDP52, and NBR1), only p62 has as yet been shown to be involved in selective degradation of peroxisomes. Since these adaptors partly share mechanistic features such as bridging ubiquitinated cargo (e.g., cytoinvasive bacteria in the case of NDP52) to LC3 family members to link with the autophagy machinery, it is unclear to date how cargo selectivity is facilitated in mammals. An interesting finding on this theme comes from the field of xenophagy, the selective degradation of cytosolic pathogens (reviewed elsewhere in this special issue): As shown recently, the two ubiquitin-binding autophagy adaptors p62 and NDP52 are recruited independently to cytoinvasive *Salmonella sp*. and show distinct localization signals at the surface of the invaded pathogens [126]. The authors argue that two individual adaptor complexes are required for effective xenophagy of *Salmonella sp*. and that these two complexes organize distinct microdomains associated with bacteria. With respect to pexophagy, it has not been analyzed whether or not different adaptor proteins are involved in selective degradation of peroxisomes and what their respective contribution is. Answers to this type of question will be informative not only for pexophagy, but for the whole field of selective autophagy pathways. A hypothetical mechanistic model of mammalian pexophagy is illustrated in Figure 7.

## 12. Pexophagy in Plant and Human Pathogens

A very interesting and unexpected perspective originates from recent studies in the field of parasitology and infection biology showing that pexophagy is required for the phytopathogenicity of the cucumber anthracnose fungus, *Colletotrichum orbiculare* [127, 128]. This plant pathogen forms a specific structure termed the appressorium, which is required for penetration of the host epidermal cells in the course of infection. The authors used a random insertional mutagenesis screen to identify fungal genes that contribute to pathogenicity. They identified the *C. orbiculare* ortholog of *P. pastoris ATG26* to be essential for host cell infection. *PpATG26* is a well-characterized pexophagy gene encoding a sterol glucosyltransferase, which is essential for pexophagy in methylotrophic yeasts [46, 92]. In the case of *C. orbiculare*, the pathogen undergoes morphological changes reminiscent of pexophagy during development of its appressoria as indicated by vacuolar localization of peroxisomes and the requirement for the central autophagy protein, Atg8. While appressoria could still be formed in the *atg26* deletion mutant, the infection process was significantly delayed. Moreover, deletion of *atg8* completely abolished appressoria formation, suggesting an essential role of the autophagy machinery during infection. As the authors show, nonselective general autophagy is essential for early morphogenesis during pathogen development, while Atg26-dependent selective pexophagy is essential for later stages of direct host-pathogen infection steps. The authors conclude that Atg26-mediated pexophagy might be involved in maturation of the infection structures by providing molecular building blocks through organelle recycling.

Another report points to the role of pexophagy during developmental and environmental changes in the parasitic protozoan, *Trypanosoma brucei*. This human pathogen, which causes sleeping sickness and Chagas disease, harbors essential enzymes of glycolysis in its peroxisomal structures, which are therefore referred to as “glycosomes.” The parasitic life cycle of this pathogen, which comprises different developmental stages in the Tse-Tse fly vector and the human host, requires adaptation of its metabolism to the changing environment. The necessary dynamic remodeling of glycosomal structures is facilitated by fusion of glycosomes with acidic lysosomes through autophagy-related mechanisms resembling pexophagy [129]. As shown recently, the acidic pH of the lysosomal compartment is responsible for inactivation of the key peroxisomal enzyme, Hexokinase (TbHK1), without affecting its protein level [130]. In addition, the pH change renders the enzyme sensitive to metabolic feedback regulation by both its substrate and product (ATP and ADP, resp.) and to modulation by other glycosomal metabolites, most likely by subtle changes in the protein tertiary structure. Thus, pexophagy appears to allow for a novel mechanism of regulating enzymatic activity by facilitating pH-dependent structural changes and concomitant feedback responses. These data point to an unexpected role of pexophagy as a regulator of essential enzyme activity in a parasitic protozoan during development and adaptation.

Moreover, the human fungal pathogen, *Candida glabrata*, requires adjustment of peroxisome number for survival after phagocytosis by immune cells [131]. The authors used fluorescent fusion proteins of transcription factors and peroxisomal enzymes to assess the metabolic status of the engulfed parasite. Using this approach, they showed that the pathogen responds to phagocytosis by increasing peroxisome number initially, most likely to fight phagocyte-induced oxidative stress. However, prolonged phagocytosis resulted in carbon starvation and a pexophagy-mediated decrease of peroxisomes. The requirement of this mechanism was shown by the dramatic loss in parasite survival during phagocytosis when the selective autophagy gene, *CgATG11*, or the general autophagy gene, *CgATG17*, were knocked out. The authors conclude that autophagy-related mechanisms, including pexophagy, represent important survival mechanisms for *Candida* after engulfment by phagocytes, pointing to the pivotal role of these pathways for providing essential cellular resources.

Kawaguchi et al. (2011) recently reported on a possible physiological role of pexophagy in yeast. This was achieved by exploring the relationship between the methylotrophic yeast *Candida boidinii* and the phyllosphere of growing *Arabidopsis thaliana* leaves [132]. The authors developed a methanol sensing assay in live *C. boidinii* cells using a PTS1-tagged fluorescent protein expressed from a methanol-inducible promoter, whereby an increase in environmental methanol concentrations resulted in enhanced fluorescence levels. They then used this assay to measure local methanol concentrations at the phyllosphere of growing* A. thaliana* leaves and showed that methanol concentrations at the phyllosphere change throughout the day corresponding to the light-dark cycle, whereby methanol concentration increased in the dark period, compared to the light period. In addition, they showed that autophagy as well as pexophagy are both required for yeast growth and survival at the phyllosphere, as autophagy and pexophagy mutants exhibited impaired proliferation on growing *A. thaliana* leaves. These results reveal interesting mechanisms used by methylotrophic yeast to survive at the phyllosphere, and how both autophagy and pexophagy are used to adapt to changes in environmental methanol dynamics, providing insight into plant-microbe interactions.

The common conclusion of the studies mentioned above is that pexophagy represents an important mechanism for survival and development under changing environmental conditions. Peroxisomes represent highly dynamic structures: Their biomass can easily be increased when peroxisomal functions are needed for specialized metabolic pathways or breakdown of damaging ROS, but they are quickly recycled when conditions change and they are not essential, so molecular building blocks and energy resources can be provided for alternative cellular functions.

Taken together, these studies point to a pivotal role of pexophagy in the development and morphogenesis of important plant and human pathogens.

## 13. Future Perspectives

Despite the great achievements of the last decade with respect to unraveling the molecular mechanisms contributing to pexophagy in various organisms, several aspects still remain to be resolved. The physiological role of pexophagy in model organisms such as yeast cells is still a matter of debate. With few exceptions, knockout of genes specifically involved in yeast pexophagy does not necessarily result in reduced viability or increased cell death. In fact, the role of pexophagy may rather be associated with quick adaptation to changing environmental conditions and thus may only emerge under cellular stress conditions like nitrogen starvation after growth under peroxisome proliferation conditions. In line with this view, pexophagy in other organisms appears to play a role for removal and recycling of unwanted or nonessential peroxisomes under condition when the cell is in need of molecular building blocks for alternative pathways, for example, for vital morphogenesis and development. In addition, the role of peroxisome turnover and linked changes in cellular redox state with cellular aging processes is increasingly recognized and warrants further investigation.

Although recent advances have pointed to a formerly unrecognized role of specific signal transduction pathways for the regulation of pexophagy (and mitophagy), the molecular framework of this process still remains to be elucidated. Which upstream events activate MAPKs differentially? Which are the pivotal targets of the protein kinase activity in this context, and how do these contribute to pexophagy regulation? Is there a molecular link between mitophagy and pexophagy? Moreover, it is not known if this mechanism is restricted to yeasts or if other organisms share the same regulatory circuits and if they have functional homologs of all the selectivity factors. Future work will therefore focus on elucidating the underlying conserved (or distinct) mechanisms.

The identity and mode of action of autophagy adaptors for yeast pexophagy is one major aspect of current research efforts. While Atg30 has been identified as an Atg11-binding pexophagy adaptor, the identity of the (proposed) Atg8-binding partner remains unresolved. In addition, while the requirement of phosphorylation for adaptor protein binding to Atg11 is well established [34, 96], it has not been addressed yet to what extent other posttranslational modifications, such as ubiquitination and/or alternative processing, of adaptor proteins contribute to the execution of pexophagy. Indeed, phosphorylation events in close proximity to Atg8-interaction motifs (AIMs) or LC3-interacting regions (LIRs) in the mammalian adaptor protein optineurin (OPTN) have been suggested as an important regulatory mechanism in xenophagy [133]. Unraveling the corresponding mechanism in yeast and mammalian pexophagy therefore represents an intriguing perspective.

The origin of membrane material for the autophagosome/phagophore membrane is still an unanswered question. Several current models argue for a contribution of the ER to provide membrane lipids and structural components. Sar1, an ER protein required for the secretory pathway, has been shown to have a role in pexophagosome formation, but the data do not unambiguously show that the pexophagosome membrane derives from the ER [70]. In addition, we have previously shown that Atg17 trafficks from the peripheral ER and colocalizes with Atg35, which regulates MIPA formation [43] but there is not a decisive mechanism of membrane trafficking as of yet.

In addition, the subcellular sorting mechanisms, which would be required to facilitate this process of membrane recruitment, are largely unknown yet recent advances towards membrane expansion and the requirement of SNAREs for autophagosome formation provide some insights [134]. However, we still need to understand how various membrane fusion events are orchestrated during pexophagy.

The role of cytoskeleton components for pexophagy is not yet fully understood. While pexophagy in yeast cells requires the actin skeleton [112], it appears that pexophagy in mammalian cells is dependent on tubulin-mediated interaction of LC3 family members with peroxisomal membrane proteins such as Pex14 [119]. Another form of selective autophagy, xenophagy of intracellular *Listeria *and *Salmonella*, relies on components of the actin skeleton, a process mediated by the increasingly characterized class of septin proteins [135, 136]. Further work is needed to decipher the contribution of cytoskeleton elements and septins for different selective autophagy pathways.

While present studies have focused on experimental systems wherein pexophagy is induced by peroxisome proliferation followed by different starvation conditions, it will be a challenging task to analyze shared and distinct mechanisms for the degradation of damaged peroxisomes. Recent experiments have provided inroads to examine damage-induced pexophagy by destabilization of peroxisome membrane proteins [71]. Whether or not this interesting finding relates to physiological processes, and if the same mechanism is conserved in other yeasts and higher eukaryotes, remains to be unraveled.

Given the emerging link between peroxisome biology and the infection cycle of important viral pathogens (e.g., HIV, influenza, and rotavirus) on the one hand, and the contribution of peroxisomes to viral detection and innate immune responses on the other hand [137, 138], it will be of utmost importance to define the role of pexophagy in the context of these important human pathogens.

Future work will shed light on these and other unanswered questions addressing the molecular basis of peroxisome turnover pathways. The resulting insights are estimated to further our understanding of selective autophagy in general.

## Figures and Tables

**Table 1:** Genes involved in macro- and micropexophagy in methylotrophic yeasts. Involvement of the respective genes in the different modes of pexophagy is indicated by check marks. Genes denoted in bold font are (by current knowledge) exclusively involved in pexophagy, but not in other autophagy pathways. Genes denoted in regular font represent components of the core machinery involved in different autophagy pathways in the methylotrophic yeasts *Pichia pastoris (Pp) *and *Hansenula polymorpha (Hp)*. Empty spaces and parentheses depict the current lack of conclusive evidence. Table adapted from Sakai et al. [55].

Gene	Description of molecular events	Macropexophagy		Micropexophagy	Reference
		*Pp*	*Hp*	*Pp*	
*ATG1*	Serine/threonine kinase required for PAS formation	*✓*	*✓*	*✓*	[42, 56, 57]
*ATG2*	Peripheral membrane protein required for Atg9 recycling	*✓*		*✓*	[58]
*ATG3*	E2-like ubiquitin ligase that catalyzes lipidation of Atg8			*✓*	[59]
*ATG4*	Protease that processes Atg8 as prerequisite for conjugation with phosphatidylethanolamine (PE)			*✓*	[60, 61]
*ATG6*	Subunit of PI3K complexes I and II	*✓*			[35]
*ATG7*	E1-(ubiquitin activating enzyme)-like protein involved in conjugation of Atg12-Atg5 and Atg8-PE conjugates	*✓*		*✓*	[42, 62]
*ATG8*	Ubiquitin-like protein that is anchored to the expanded phagophore membrane in its processed and lipidated form, involved in phagophore membrane expansion	*✓*	*✓*	*✓*	[33, 60, 61]
*ATG9*	Transmembrane protein cycling between the PAS and a peripheral compartment	*✓*		*✓*	[57, 63]
*ATG11*	Coiled-coil adaptor protein that interacts with the core machinery and known receptors for selective autophagy	*✓*	*✓*	*✓*	[64]
*ATG16*	Essential component of the Atg12-Atg5-Atg16 complex	(*✓*)		*✓*	[42]
*ATG17*	Scaffold protein that is responsible for PAS organization	*✓*		*✓*	[34]
*ATG18*	PtdIns3P-binding protein whose localization is dependent Atg9 and PtdIns-3P; recruits Atg2 and needed for Atg9 recycling	*✓*		*✓*	[65, 66]
*ATG21*	WD40 protein with phosphoinositide binding domain that is involved in pexophagosome formation		*✓*		[67]
***ATG24***	Sorting nexin protein involved in fusion events with the vacuole	*✓*		*✓*	[33]
***ATG25***	Coiled-coil protein that co-localizes with Atg11 at the PAS, required for macropexophagy		*✓*		[39]
***ATG26***	Sterol glucosyltransferase that plays a role in phagophore membrane expansion	*✓*		*✓*	[44, 46, 68]
***ATG28***	Coiled-coil protein required for peroxisome sequestration during micropexophagy and vacuole fusion of pexophagosomes in macropexophagy	*✓*		*✓*	[69]
***ATG30***	Pexophagy receptor that interacts with peroxins, Pex3 and Pex14, and adaptor proteins, Atg11 and Atg17	*✓*		*✓*	[34]
***ATG35***	Localizes to the perinuclear structure; regulates MIPA formation and interacts with Atg28 and Atg17			*✓*	[43]
*GCN1-4*	Involved in general amino acid control			*✓*	[42]
***Sar1***	Sec protein required for MIPA and proper pexophagosome formation		*✓*	*✓*	[70]
*PEP4*	Vacuolar protease			*✓*	[60]
***PEX3***	PMP peroxin required for peroxisome biogenesis and for recruitment of pexophagy receptor	*✓*	*✓*	*✓*	[34, 71, 72]
***PEX14***	PMP peroxin required for peroxisome biogenesis and for recruitment of pexophagy receptor	*✓*	*✓*	*✓*	[34, 73]
***PIK1***	PtdIns-4-kinase required for MIPA formation			*✓*	[46]
***PFK1***	Subunit of phosphofructokinase complex			*✓*	[62]
*TUP1*	Transcriptional repressor	*✓*			[74]
*VAC8*	N-myristoylated armadillo-repeat protein of the vacuolar membrane, required for VSM formation			*✓*	[75, 76]
*VAM7*	SNARE protein that is involved in vacuolar fusion events with the phagophore membrane		*✓*		[45]
*VPS15*	Regulatory subunit of PI3K	*✓*	*✓*	*✓*	[42, 77]
*VPS34*	Phosphatidylinositol-3-kinase (PI3K)	*✓*	*✓*	*✓*	[36, 78]
*YPT7*	Rab GTPase involved in phagophore membrane fusion	*✓*	*✓*	*✓*	[71, 79]

## Abbreviations

15-LOX: 15-Lipoxygenase
3-MA: 3-Methyladenine
4-PBA: 4-phenylbutyrate
AIM: Atg8-Interaction Motif
ATG: Autophagy-related
CHO: Chinese Hamster Ovary cells
Cvt: Cytosol-to-Vacuole Targeting
DEHP: Di-(2-ethylhexyl) phthalate
FYCO1: FYVE and coiled-coil domain-containing 1 protein
GRAM: Domain found in glycosyltransferases, Rab-like GTPase activators and myotubularins
MAPK: Mitogen Activated Protein Kinase
MIPA: Micropexophagy-specific membrane Apparatus
PAS: Phagophore Assembly Site
PBD: Peroxisomal Biogenesis Disorders
PD: Peroxisomal Disorders
PE: Phosphatidylethanolamine
PED: Peroxisomal Enzyme/Transporter Deficiencies
PEX: Peroxin
PI3K: PtdIns-3-kinase
PM: Phagophore Membrane
PMP: Peroxisomal Membrane Protein
PPAR: Peroxisome-Proliferator Activated Receptor
PtdIns3P: phosphatidylinositol-3-phosphate
PtdIns4P: phosphatidylinositol-4-phosphate
PTS: Peroxisomal Targeting Signal
PVS: perivacuolar structures
PX: PtdIns3P-binding module
ROS: Reactive Oxygen Species
TbHK1: *Trypanosoma brucei* peroxisomal Hexokinase
VSM: Vacuole Sequestering Membrane.
